# Possible Effects of Proton Pump Inhibitors on Hearing Loss Development

**DOI:** 10.1155/2019/4853695

**Published:** 2019-10-17

**Authors:** Michał Wiciński, Bartosz Malinowski, Oskar Puk, Karol Górski, Dawid Adamkiewicz, Grzegorz Chojnacki, Maciej Walczak, Eryk Wódkiewicz, Monika Szambelan, Paulina Adamska, Kamila Skibińska, Maciej Socha, Maciej Słupski, Katarzyna Pawlak-Osińska

**Affiliations:** ^1^Department of Pharmacology and Therapeutics, Faculty of Medicine, Collegium Medicum in Bydgoszcz, Nicolaus Copernicus University, M. Curie 9, 85-090 Bydgoszcz, Poland; ^2^Department of Obstetrics, Gynecology and Gynecological Oncology, Faculty of Medicine, Collegium Medicum in Bydgoszcz, Nicolaus Copernicus University, Ujejskiego 75, 85-168 Bydgoszcz, Poland; ^3^Department of Hepatobiliary and General Surgery, Faculty of Medicine, Collegium Medicum in Bydgoszcz, Nicolaus Copernicus University, M. Curie 9, 85-090 Bydgoszcz, Poland; ^4^Department of Pathophysiology of Hearing and Balance System, Collegium Medicum, Nicolaus Copernicus University, Bydgoszcz, Poland

## Abstract

Considered safe and often available as over-the-counter (OTC) drugs, proton pump inhibitors (PPI) are one of the most frequently used medicines. Over recent years much research analyzing PPI has been conducted and these studies shed light on PPI side effects and the mechanisms of these processes. In this study we summarize the findings of these studies and through deduction present some hypotheses on the impact of PPI on health. Of particular interest is the impact of PPI on hearing loss development. However, despite this side effect being localized, its mechanisms are complex, systemic and involve changes in whole body. This paper summarizes how through, inter alia, alterations in the circulatory system, respiratory system, central nervous system and metabolism PPI can cause hearing impairment, which can occur in every age group and is connected with long-term use of this group of drugs. This article also discusses the role PPI plays in the acceleration of presbycusis development, in relation to the fact that older people are the group who most frequently use PPI in long term. Hearing loss negatively impacts affects quality of life, especially among older patients who are also the most afflicted group; administration of PPI should therefore be considered carefully, taking into consideration all potential benefits and side effects.

## 1. Introduction

Proton pump inhibitors (PPI) are one of the most commonly used drugs around the world, second in usage only to statins. They are a group of drugs commonly used as a standard therapy in gastroesophageal reflux disease (GERD) and acidity disorders of the upper gastrointestinal tract. Due to their proven effectiveness in suppression of acid secretion by gastric parietal cells [1], PPI are used both in the treatment and the prevention of gastric and duodenal ulcers, gastroesophageal reflux disease and in the eradication of* Helicobacter pylori*. Their ubiquitous use is also due to the administration of PPI to patients receiving non-steroidal anti-inflammatory drugs or antiplatelet agents [2, 3]. In addition to the well-known use in treatment of inflammation of the upper gastrointestinal tract, the number of alternative PPI applications is constantly increasing, including the treatment of a variety of respiratory symptoms, sleep disorders, as well as hypersensitivity and hyperactivity in children [4–7]. Some clinicians state that PPI are too frequently prescribed in situations when they are not required, and excessive use of this group of drugs can lead to increased occurrence of side effects, especially if they are used for a long time. This work aims to gather research reports/studies and to define the impact of PPI on hearing and their potential role in hearing loss development.

## 2. Findings

### 2.1. Otitis Media and Upper Respiratory Tract Infections

Although PPI are generally considered safe, concerns are now growing about the safety of these medicines, especially among children [8]. Changes in the human body caused by PPI may include: dysbiosis, local mucosal secretory alterations, bacteria functional and morphological changes, and other potential factors that may contribute to the body's dysfunction [9]. The most dangerous results of those changes are upper respiratory tract infections and possibly otitis media [9].

Changes in the microbiome play a special role in the formation of otitis media. The relation between the use of PPI and changes in the microbiome is crucial for understanding the mechanism of PPI side effects associated with inflammation and others [9]. An article by Rosen et al. demonstrated that quantitative changes in the microbiome related to PPI not only concern the gastric microbiome, but also the lung and oropharyngeal microbiome. This dependence applies to the altered microbiome, disturbance of the gastric acid barrier, local bacterial overgrowth, and direct effects on bacteria; all of these factors increase the risk of infection [9]. The mechanism of altering the microbiome by PPI is based on the weakening of immune cell activation, migration and function [10]. PPI also affect epithelial cell signaling by inhibiting transcription of IL-8, thus impairing immunological response to microorganisms [11]. Administration of omeprazole in standard doses results in irreversibly reduced neutrophil chemotaxis and inhibits oxygen derived free-radical generation, which may also induce degranulation [12]. In vitro, PPI inhibit human neutrophil H^+^/K^+^ ATPase activity, which leads to inhibition of cell migration and causes intracellular calcium influx [13]. Moreover, PPI may reduce polymorphonuclear leukocyte chemotaxis, as well as suppress the mitogen-activated protein kinase transduction signal and inhibit cytokine production [14].

PPI can reduce the effectiveness of antibiotics by acting on bacterial proteins or biological pumps. Interestingly, in an in vitro study, addition of omeprazole, pantoprazole or lansoprazole to a bacterial isolate containing tigecycline resulted in an increase of the median inhibitory concentration by more than 128-fold [15]. In an area of invasive infection by microorganisms facilitated by PPI, a local inflammatory reaction may occur, creating a microenvironment that promotes additional pathogenic bacterial colonization, which further increases the risk of disease [16].

A retrospective study of 102 patients suffering from liver cirrhosis suggests that PPI promotes the translocation of bacteria through the intestinal epithelium and stomach [17]. There is increasing evidence that PPI influences changes in the microbiome, secretion and antibacterial properties of mucous surfaces, including those outside the gastrointestinal system [18, 19].

Some pharmacological studies suggest that PPI may have effects at the cellular and systemic level in addition to inhibition of acid secretion. PPI may exacerbate symptoms of postnasal drainage (excessive mucus production syndrome) regardless of the symptoms of reflux and present pH of the esophagus [20]. Nasal microvasculature and paranasal sinuses play an integral role in maintaining health. Dysbiosis is a possible cause of chronic rhinosinusitis and other upper respiratory tract diseases [21]. Alterations to the microbiome, local changes in mucus secretion and inflammation may predispose children to upper respiratory tract infections [9]. Recent studies have established that children with recurrent acute otitis media and chronic otitis media with effusion have different nasopharyngeal flora and bacterial resistance compared to healthy children [22]. A study involving 271 children with asthma showed that the group of children with poor metabolism of the specific cytochrome P450 2C19 haplotypes had higher rates of upper respiratory tract infections after administration of lansoprazole when compared to the placebo group, OR 2.46 (95% CI: 1.02–5.96) [23]. Moreover, even among children with high activity of CYP2C19, infections occurred more frequently than in the placebo group, OR 1.55 (95% CI: 0.86–2.79) [23]. These results suggest that side effects of PPI may be aggravated by the CYP2C19 haplotype. Changing a single nucleotide of the CYP2C19 gene may delay the removal of the drug, which will prolong exposure to the drug, increasing the probability of occurrence of side effects [24]. More precise studies that show the relationship between PPI and sinusitis or middle ear inflammation are therefore still required.

A systematic review conducted by de la Coba Ortiz et al. showed that use of PPI is associated with a greater risk of enteric infections, OR 3.33 (95% CI: 1.84–6.02), as well as pneumonia, OR 1.49 (95% CI: 1.16–1.92). Moreover, treatment time of patients taking PPI was significantly elongated, with odds ratio for more than one month of treatment of 2.10 (95% CI: 1.39–3.16) and neither dose nor patient age had an influence on this effect. Furthermore, these infections were associated with more hospitalizations, OR 1.61 (95% CI: 1.12–2.31) [25].

There are several major studies revealing a significant positive correlation between otitis media in childhood and adult hearing loss, reporting reduced adult hearing thresholds in the entire frequency range if such infections occurred [26, 27]. Tian et al. designed a study to assess morphological and histological alterations in the middle ear during the course of otitis media with effusion [28]. In this study, researchers examined mice with a point mutation in the Enpp1 gene, which resulted in a high incidence of otitis media. Evaluation at 12 weeks old showed hearing loss among 90% of the mutant mice, which was connected with otitis media with effusion. The morphological and histological alterations found in samples obtained from mice with hearing loss were: increased amount of inflammatory cells, thickened middle ear epithelium with fibrous polyps, increased amount of mucin-secreting goblet cells, over-ossification at the round window ridge, thickened and over-calcified stapedial artery, fusion of malleus and incus, and white patches on the inside of a tympanic membrane, some of which are typical symptoms of tympanosclerosis [28].

All of the abovementioned findings found a correlation between otitis media and hearing loss, and revealed a possible pathomechanism of that process.

### 2.2. Vascular Disorders after PPI Usage

**Figure 1:**
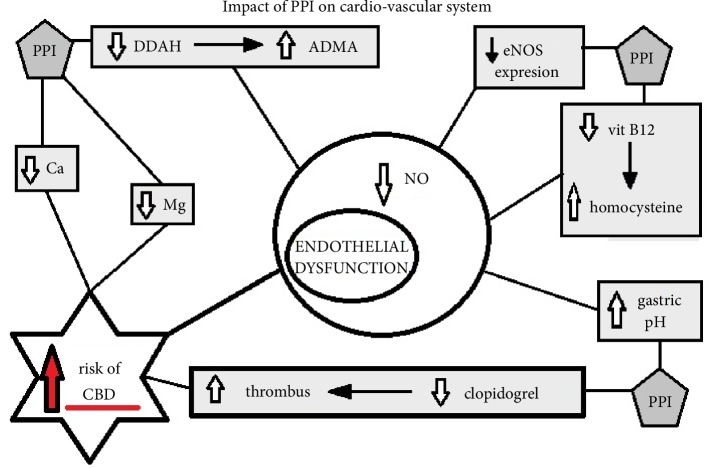
Impact of PPI on cardio-vascular system. DDAH, dimethylaminohydrolase; ADMA, asymmetric dimethylarginine; eNOS, endothelial nitric oxide synthetase; CBD, cochlear blood-flow disorders.

PPI are connected with several side effects, which appear particularly after long-term usage and one of the most dangerous, undesirable actions shown by the conducted research is the unfavorable effect of PPI on vascular homeostasis and the increased risk of major adverse cardiovascular events. Large clinical studies have shown that PPI use is associated with myocardial infarction, OR 1.16 (95% CI: 1.09–1.24), and this adverse effect is independent of interaction with clopidogrel [25]. The cochlea is provided with blood only by the labyrinthine artery and as such it is very susceptible to ischemia, during which degeneration of the outer and inner hairy cells occurs [29–33]. Vascular pathomechanisms are presented in Figure 1.

The most important and well-known mechanisms causing endothelial dysfunction are interdependent pathways that decrease the vascular concentration of nitric oxide (NO) [29, 34]. It has been demonstrated that PPI may have an adverse effect on the cardiovascular system by reducing the activity of endothelial nitric oxide synthetase (eNOS) [34]. eNOS provides a constant amount of nitric oxide (NO) in the vessels, independent of inducing factors. High biological activity of NO is a key element in the regulation of vascular homeostasis, maintaining anticoagulant activity, i.e. vasodilatation and reduction of platelet adhesion and aggregation [35], as well as having an anti-atherosclerotic and anti-inflammatory effect [36–38]. Numerous epidemiological studies have shown that patients with impaired eNOS activity are more exposed to major adverse cardiovascular events (MACE) [39]. PPI directly inhibit the expression of endothelial NOS proteins [40]. Moreover, it has been proven that PPI indirectly inhibit NOS by means of a circulating endogenous enzyme reducing factor—asymmetric dimethylarginine (ADMA). ADMA arises from the catabolism of cellular proteins containing methylarginine residues and is eliminated by dimethylarginine dimethylaminohydrolase (DDAH) [41]. It is known that the main reason for the increase in ADMA concentration is a decrease in its degradation by the binding and inhibiting degrading enzyme (DDAH) caused by PPI. An elevated ADMA concentration disturbs vascular homeostasis and weakens vascular protective actions, and is therefore a potential risk factor for morbidity and mortality from cardiovascular causes [42–44], which was confirmed in clinical trials of patients with ACS [45]. It is worth noting that the concentration of DDAH inhibitors is similar in all PPI [34], and the increase in circulating ADMA can be observed after only one week of PPI usage [40]. At this stage, it is worth showing that the main known factor directly decreasing the concentration of DDAH and indirectly increasing the concentration of circulating ADMA is hyperhomocysteinemia [46]. The destructive effects of elevated homocysteine in the blood on endothelial cells are related to the interaction of homocysteine thiol groups, which may contribute to an increase in the amount of oxygen free radicals, which damage the intrinsic layers of blood vessels [47, 48]. This amino acid, homocysteine, inhibits the expression and activity of DDAH that catalyzes the breakdown of the endogenous inhibitor of nitric oxide synthase, ADMA. By decreasing vascular flexibility and impairing diastolic function of vessels, homocysteine shows atherogenic properties. This is confirmed through ultrasound examination, in which the degree of atherosclerosis in blood vessels is correlated with homocysteine concentration in blood samples of patients [49]. Moreover, this amino acid directly reacts with nitric oxide to give S-nitrohomocysteine, which also reduces the concentration of free nitric oxide. In addition, it disturbs the process of hemostasis by activating coagulation factors V, VII and XII, simultaneously reducing the binding capacity of tissue plasminogen activator and the activity of C protein [50–52]. Hyperhomocysteinemia has been shown to be largely caused by the disturbance of homocysteine to methionine transformation, a process that is impaired by a deficiency of vitamin B12 (cobalamin), which is a catalyst for this reaction. PPI contribute to the disturbance of that process and cause hyperhomocysteinemia. There is a hypothesis explaining the mechanism of this disorder, according to which the use of PPI probably reduces the absorption of vitamin B12 by inhibiting intragastric proteolysis, thus preventing the release of vitamin B12 from the food and combining with Castle's factor [53]. It turns out that PPI are in conflict with both the standard mechanism of NO production and the new, alternative route for formation of nitric oxide: nitrate → nitrite → nitric oxide. In this alternative route, mouth flora transform nitrate to nitrite, which when swallowed goes to the acidic environment of the stomach. There, nitrite (nitric acid) can be reduced to nitric oxide (NO) due to the low pH and increase the vasodilatation effect created by NO. This conversion is supported by the antioxidant effect of vitamin C (ascorbic acid). The antioxidant action of ascorbic acid promotes the conversion of nitric acid to NO instead of to inactive N-nitroso compounds and protects NO from degradation. It is likely that administration of PPI increases the gastric pH and both inhibits the last reaction of the alternative pathway, non-enzymatic production of NO, and impairs the absorption of ascorbic acid. To confirm this hypothesis researchers administrated a hypotensive sodium nitrite in combination with PPI (omeprazole) to patients. The study showed that omeprazole can decrease the anti-hypertensive effect exerted by sodium nitrite administered orally, but not intravenously [53–55].

Another possible mechanism through which PPI may increase the risk of MACE occurrence was discovered during the study of new antiplatelet drugs. The explanation for this mechanism of interaction is based on cytochrome CYP2C19, which is necessary for the activation of clopidogrel, as well as the metabolism of PPI, which results in competitive inhibition. In particular, PPI with high affinity for CYP2C19, such as omeprazole and esomeprazole, may reduce the antiplatelet efficacy of clopidogrel. Clinical trials describing the relationship between clopidogrel and PPI and cardiovascular risk are ambiguous. There are studies showing an increased incidence of MACE when patients took PPI while on clopidogrel [56–58]. On the other hand, there are also studies in which MACE risk was independent of whether the patient was using PPI or PPI in combination with clopidogrel [59, 60]. A systematic review by de la Coba Ortiz at al. showed that PPI significantly decrease platelet function inhibition, (39.8 ± 15.4% versus 51.4 ± 16.4%, p < 0.0001, percentage of inhibited platelets, omeprazole vs. placebo respectively) [25]. These results, together with concerns about the outcome of possible side effects, led to the issuing of a warning by the American Food and Drug Administration (FDA) and the European Medicines Agency (EMA) regarding the use of PPI with clopidogrel [61, 62].

All of the abovementioned possible complications that impair vasodilatation and limit the vascular flow are a consequence of a microcirculation impairment, suppression of anticoagulation mechanisms and prothrombotic activation, and can lead to embolization of vessels.

There are reports of hearing loss in which pathophysiology is based on the distortion of blood flow in the cochlea [63, 64]. However, due to the relatively small dimensions, delicacy and deep position in the temporal bone, the human cochlea is not easily available for examination. These researchers report the positive correlation between hearing loss and atherosclerosis [65], basing this on the degeneration of the cochlea caused by atherosclerosis in young adults, in whom loss of outer hair cells in the cochlea can occur due to impaired perfusion [66]. However, some research reports preserving good local perfusion despite the generalized atherosclerotic process, concluding that there is no correlation between hearing loss and atherosclerosis [67]. However, Mazurek et al. [67] established that hypoxia, regardless of the underlying cause, resulted in a mean loss of 8% of outer hair cells and 14% of inner hair cells after 8 h exposure. Moreover, ischemia resulted in a mean loss of 19% of outer hair cells and 39% of inner hair cells after 8 h exposure [68]. Further research is needed to fully assess the effect of vascular disorders on the hearing impairment.

### 2.3. Metabolic Changes

**Figure 2:**
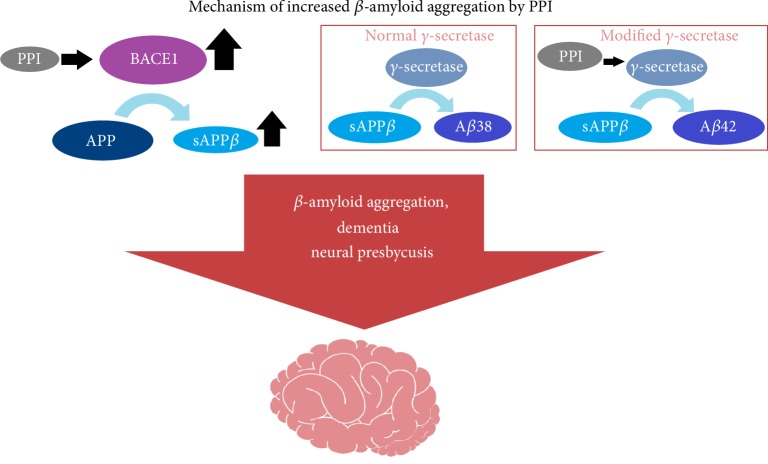
Mechanisms of increased *β*-amyloid aggregation by PPI. APP, Amyloid Precursor Protein; sAPP*β*, soluble Amyloid Precursor Protein beta; BACE1, beta-site amyloid precursor protein cleaving enzyme 1.

Recent studies have shown that PPI use can lead to an increase in the level of *β*-amyloid in the brain and that dementia strongly correlates with hearing loss via the mechanism of neural presbycusis [69–71]. Estimated hazard ratios of dementia and Alzheimer's disease in course of treatment with PPI are 1.38 (95% CI: 1.04–1.83) and 1.44 (95% CI: 1.01–2.06), respectively [25]. Despite the fact that the mechanism of *β*-amyloid aggregation has not yet been confirmed, we can find two promising hypotheses in the available literature [70, 71]:

1. Modulation of a gamma secretase cleavage site and increase of the beta-secretase 1 (BACE1) and other pH dependent proteases activity (Figure 2);
2. Decreased degradation by lysosomes in microglia due to inhibition of vacuolar type H^+^-ATPase leading to increased pH and reduced clearance of *β*-amyloid peptides.

These mechanisms are not mutually exclusive and it is quite possible that both of them occur. The first hypothesis explains the increased levels of amyloid *β*-peptide 37 (A*β*37), amyloid *β*-peptide 40 (A*β*40) and amyloid *β*-peptide 42 (A*β*42) fractions and the decreased level of the amyloid *β*-peptide 38 (A*β*38) fraction. Badiola et al. [70] discovered that the effect of lansoprazole and other PPI elevate *β*-amyloid level in a dosage-dependent fashion, but do not describe the long-term-use effect. Although the most significant elevation was observed in high-dosage models, elevated levels were also seen in models with commonly used concentrations [70]. Dementia and neural presbycusis lead to a severe decrease in speech discrimination, which is particularly onerous for patients and is a major indicator for qualification for hearing aid use [69]. It is important to consider that increased amyloid level occurs not only in Alzheimer's disease but also in most cognitive impairment states; we can therefore infer that this problem may be underestimated and wider studies with more complex models are necessary to obtain an understanding of the full scope of the problem [72, 73].

PPI alter absorption of certain nutrients such as vitamin B12, Fe and NO [34, 71, 74]. Vitamin B12 deficiency is an effect of hypochlorhydria, which impairs release of dietary protein-bound vitamin B12 that can be absorbed from the terminal ileum. Hypocobalaminemia can occur after approximately twelve months of PPI usage. Vitamin B12 is crucial for conversion of homocysteine to methionine thus its deficiency is a source of homocysteinemia, which has been discussed in another part of this article [34]. Insufficiency of B12 leads to impairment of neuron function, and thus can lead to dementia and neural presbycusis as well [75, 76]. Iron deficiency appears due to a decreased reduction of ferric ions to ferrous ions, which are more bioavailable. Iron deficiency is a cause of cochlear function impairment but the mechanism is not yet fully elucidated; however, there are two plausible hypotheses [75, 76]:

1. The cochlea is supplied in blood only by the labyrinthine artery and is therefore very susceptible to ischemic damage subsequent to iron deficiency anemia;
2. Reduction of cochlear ribbon synapses which transduce signals between inner hair cells and spiral ganglion cells.

Dietary-obtained nitrate forms nitrous acid in the stomach, which can be source of NO. PPI increase the gastric pH and therefore reduce that process. Furthermore, PPI inhibits the capacity of antioxidants to convert nitrite to NO in the stomach and reduces formation of S-nitrosothiols which are a circulating reservoir of NO [34]. The impact of nitrogen monoxide insufficiency has been discussed in another part of this article.

Lecain et al. have proven that a gastric proton pump is well expressed in the inner ear, particularly in the cochlear lateral wall [77]. H,K-ATPase has been also found in the choroid plexus giving rise to the hypothesis that this protein is involved in maintaining the homeostasis of the cerebrospinal fluid and inner ear fluids. Abundant expression of H,K-ATPase in the cochlear lateral wall prompted Shibata et al. to hypothesize that a gastric proton pump is crucial for circulation of K^+^ ions from perilymph to endolymph and for the maintenance of a high concentration of K^+^ ions in endolymph as an extracellular fluid. Research has supported this hypothesis, leading to confirmation of both the expression of the gastric type of proton pump in the cochlea and its role in maintaining the inner ear potential. The inhibition of H,K-ATPase by Sch-28080 resulted in a significant decrease of endolymph potential in a dosage-dependent fashion; however the effect of omeprazole was negligible, presumably due to the need for a <5 pH for activation of benzimidazole derivatives and the actual pH of perilymph is 7.8–8.0. Although PPI may not alter the endolymph and perilymph potential in healthy individuals, it may aggravate the course of inner ear diseases in which pH disorder occurs very often, further studies are needed for better comprehension of that process [77, 78].

## 3. Conclusions

**Figure 3:**
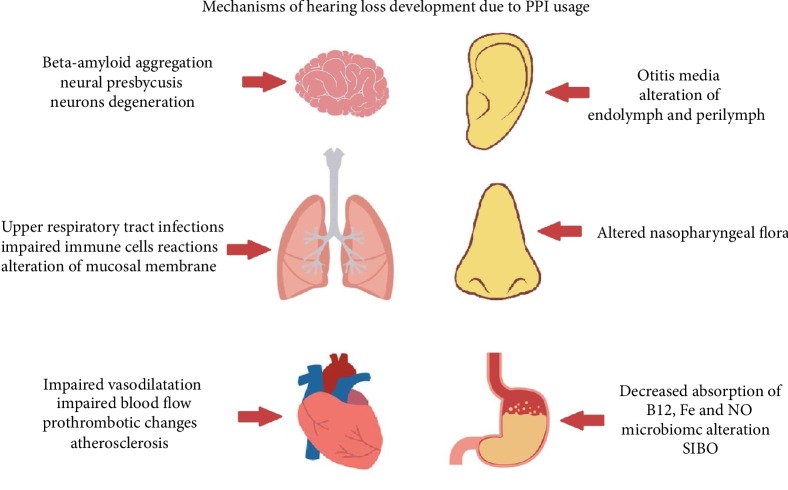
Mechanisms of hearing loss development due to PPI usage (SIBO, small intestinal bacterial overgrowth).

The mechanisms of PPI influence on hearing are complex and connected to each other (Figure 3). Moreover, hearing impairment caused by PPI can be hard to distinguish at the beginning of the process, with symptoms that concern patients only emerging after months or even years of usage of this particular group of drugs. The complexity and variety of mechanisms described above contribute to the difficulty of diagnosing hearing loss due to PPI usage and to the difficulties involved in research projecting and conducting. However there has been some research that gives us a way to view this problem, leading to the conclusion that mechanisms of PPI influence on hearing can be divided into three groups:

1. Infections and inflammatory reactions;
2. Cardiovascular disturbance;
3. Metabolic changes.

Mechanisms within the first group are the most important among pediatric patients due to the better connection between the pharynx and middle ear; recurrent otitis media or/and upper respiratory tract infections among children with prescribed PPI should therefore always concern physicians due to its potential effects on hearing. The most important pathophysiological points in this group are: altered microbiome, disturbance of the gastric acid barrier and alteration of mucosal membranes of gastrointestinal and respiratory tracts, local bacterial overgrowth, direct effect on bacteria, impaired epithelial cell signaling, impaired immune cell reactions, and decreased antibiotic effect on bacteria.

Mechanisms within the second group afflict mainly adults after forty years of age due to the increasing presence of cardiovascular problems in this group of patients. Moreover, PPI usage in this group of patients is highest due to GERD and the presence of gastrointestinal tract ulcers, therefore these patients are most highly exposed to PPI side effects. The most important pathophysiological points in this group are: decreased vascular concentration of nitric oxide, inflammatory and atherosclerotic susceptibility of blood vessels, impaired vasodilatation and blood flow, hyperhomocysteinemia, and prothrombotic changes.

The third group of mechanisms is most significant among elderly patients, although they do afflict patients in all age groups, especially vitamin B12 deficiency. Metabolic changes occur after long-term use of PPI, thus adults and elderly people are most afflicted. The most important pathophysiological points in this group are: *β*-amyloid aggregation in brain and vitamin B12 deficiency leading to neural presbycusis; iron deficiency; anemia; decreased nitric oxide level; and alteration of endolymph and perilymph.

CYP2C19 has an important role in proton pump inhibitors' unwanted effects. This enzyme metabolizes PPI, which induces many interactions, of which one of the most important is the decreased activation of clopidogrel, which impairs antiplatelet effect of this drug and significantly increases risk of major adverse cardiovascular events occurrence and impairs blood flow, inter alia, in the labyrinthine artery which directly afflicts the cochlea [56–58, 75]. Moreover, decreased activity of CYP2C19, which occurs within around 30% of the population, leads to a higher concentration of PPI and therefore an increased risk of side effects [24, 79]. All pathomechanisms are presented in Figure 3.

Mechanisms through which PPI can lead to hearing loss development can be divided not only by their pathophysiological pathways, but also by their target point; we can therefore designate mechanisms into a further three groups:

1. Middle ear alterations;
2. Inner ear alterations;
3. Cerebral cortex alterations.

Alterations of the middle ear are results of infections and inflammation and include thickening of the middle ear epithelium with fibrous polyps, increased levels of mucin-secreting goblet cells, over-ossification at the round window ridge, thickened and over-calcified stapedial artery, fusion of malleus and incus, and tympanosclerosis. These changes in the middle ear lead to conductive hearing loss as a complication of infections, and (as described earlier) PPI significantly increase the frequency of middle ear infections.

Alterations in the inner ear are the effect of various pathomechanisms such as decreased blood flow in the labyrinthine artery, prothrombotic changes that impair blood supply to the cochlea, and iron deficiency. These changes result in hypoxia of the inner ear, which leads to a reduction of the amount of outer and inner hair cells, which are crucial for sound transduction. Another alteration in the inner ear occurs due to H,K-ATPase inhibition by PPI, which leads to a reduction of electric potential of endolymph and thus impairs the function of the labyrinth. Moreover, it is possible that iron deficiency results in a reduction of cochlear ribbon synapses, which transduce signals between inner hair cells and spiral ganglion cells, thus impairing auditory system signaling.

**Figure 4:**
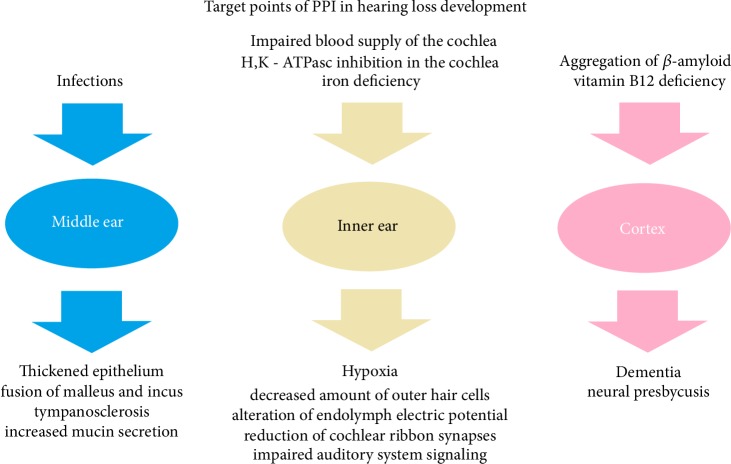
Target points of PPI in hearing loss development.

Finally, all auditory stimuli must be analyzed by the auditory cortex, and this process can be disturbed by alteration of neuronal function and a reduction of neuron number. As described earlier, this can be caused by PPI through aggregation of *β*-amyloid and vitamin B12 deficiency, leading to dementia and neural presbycusis due to impairment of auditory cortex function. All target points are presented in Figure 4.

Despite the difficulties and complexity of the problem, researchers have managed to shed some light on the role of PPI in hearing loss development. Some parts of this article are hypotheses based on deduction and connection of different alterations, for example connection of the SIBO and upper respiratory tract infections with otitis media. Although more studies focused only on the particular side effect of hearing loss are needed to develop a full and quantitative picture of this problem, the authors of this article think that PPI should be prescribed carefully and overuse of PPI as OTC drugs should be of concern to medical society.

## 4. Discussion

In analyzing the influence of PPI on hearing we could not neglect to consider the main indication for PPI usage which is GERD. Gastroesophageal reflux disease itself has been considered a risk factor for hearing loss development, mainly through otitis media which can be a complication of that disease and is an independent risk factor of hearing loss [80, 81]. Moreover, there is a study indicating a positive effect of treatment with PPI on hearing in a group of children with otitis media in course of GERD [82]. In view of these facts some researchers state that the actual risk factor is GERD and PPI are just coincidentally connected with hearing loss as a medication used for treatment of GERD [83]. The study by Lin et al. appears to confirm that GERD leads to hearing loss and PPI does not elevate that risk independently to GERD symptoms intensity. However, this study has a certain limitation [83]; all the data was gathered through a survey, including the assessment of hearing loss, which was confirmed based only on the patient's answer. This method could induce a high level of false positive and false negative results, making the following statistical analysis much less reliable. Moreover, there is no information included about dose, frequency of intake or sort of the PPI, which makes it difficult to determine for certain whether something is or is not an effect of PPI usage.

McCoul et al. decided to assess the effect of PPI treatment among young children with otitis media with effusion in the course of GERD [82]. In this study the positive effect of PPI on hearing is due to a reduction of effusion and inflammation, and appears only in acute stances. That is why the article by McCoul et al. refers only to specific cases and does not indicate long-term effects of PPI usage, which appear to be positive in acute otitis media with effusion.

Although GERD and otitis media are risk factors for hearing loss, the authors of this article, taking into consideration the abovementioned mechanisms and data, believe that PPI usage is an independent risk factor for hearing loss, especially in cases of inadequate or excessive treatment.

## References

[1] Almufleh A., Ramirez F., So D. (2018). H2 receptor antagonists versus proton pump inhibitors in patients on dual antiplatelet therapy for coronary artery disease: a systematic review. *Cardiology*.

[2] Chubineh S., Birk J. (2012). Proton pump inhibitors: the good, the bad, and the unwanted. *Southern Medical Journal*.

[3] Shi S., Klotz U. (2008). Proton pump inhibitors: an update of their clinical use and pharmacokinetics. *European Journal of Clinical Pharmacology*.

[4] Ummarino D., Miele E., Masi P., Tramontano A., Staiano A., Vandenplas Y. (2012). Impact of antisecretory treatment on respiratory symptoms of gastroesophageal reflux disease in children. *Diseases of the Esophagus*.

[5] Tofil N. M., Benner K. W., Fuller M. P., Winkler M. K. (2008). Histamine 2 receptor antagonists vs intravenous proton pump inhibitors in a pediatric intensive care unit: A comparison of gastric pH. *Journal of Critical Care*.

[6] Gieruszczak-Białek D., Konarska Z., Skórka A., Vandenplas Y., Szajewska H. (2015). No effect of proton pump inhibitors on crying and irritability in infants: systematic review of randomized controlled trials. *Journal of Pediatrics*.

[7] Hassall E. (2012). Over-prescription of acid-suppressing medications in infants: how it came about, why it’s wrong, and what to do about it. *Journal of Pediatrics*.

[8] Chung E. Y., Yardley J. (2013). Are there risks associated with empiric acid suppression treatment of infants and children suspected of having gastroesophageal reflux disease?. *Hospital Pediatrics*.

[9] Stark C. M., Nylund C. M. (2016). Side Effects and Complications of Proton Pump Inhibitors: A Pediatric Perspective. *Journal of Pediatrics*.

[10] Kedika R. R., Souza R. F., Spechler S. J. (2009). Potential anti-inflammatory effects of proton pump inhibitors: a review and discussion of the clinical implications. *Digestive Diseases and Sciences*.

[11] Huo X., Zhang X., Yu C. (2014). In oesophageal squamous cells exposed to acidic bile salt medium, omeprazole inhibits IL-8 expression through effects on nuclear factor-*κ*B and activator protein-1. *Gut*.

[12] Suzuki M., Mori M., Miura S. (1996). Omeprazole attenuates oxygen-derived free radical production from human neutrophils. *Free Radical Biology & Medicine*.

[13] Oliveira R. M., Antunes E., Pedrazzoli J., Gambero A. (2007). The inhibitory effects of H^+^K^+^ATPase inhibitors on human neutrophils in vitro: restoration by a K^+^ ionophore. *Inflammation Research*.

[14] Koshio O., Tansho S., Ubagai T., Ono Y., Nakaki T. (2010). Suppression of phosphorylation of extracellular-signal-regulated kinase and p38 mitogen-activated protein kinase in polymorphonuclear leukocytes by the proton pump inhibitor lansoprazole. *Journal of Infection and Chemotherapy*.

[15] Ni W., Cai X., Liang B. (2014). Effect of proton pump inhibitors on in vitro activity of tigecycline against several common clinical pathogens. *PLoS ONE*.

[16] Winter S. E., Winter M. G., Xavier M. N. (2013). Host-derived nitrate boosts growth of E. coli in the inflamed gut. *Science*.

[17] de Vos M., De Vroey B., Garcia B. G. (2013). Role of proton pump inhibitors in the occurrence and the prognosis of spontaneous bacterial peritonitis in cirrhotic patients with ascites. *Liver International*.

[18] Hegarty J. P., Sangster W., Harris L. R., Stewart D. B. (2014). Proton pump inhibitors induce changes in colonocyte gene expression that may affect Clostridium difficile infection.. *Surgery*.

[19] Lameris A. L., Hess M. W., van Kruijsbergen I., Hoenderop J. G., Bindels R. J. (2013). Omeprazole enhances the colonic expression of the Mg^2+^ transporter TRPM6. *Pflügers Archiv - European Journal of Physiology*.

[20] Vaezi M. F., Hagaman D. D., Slaughter J. C. (2010). Proton pump inhibitor therapy improves symptoms in postnasal drainage. *Gastroenterology*.

[21] Sieczkowska A., Landowski P., Zagozdzon P., Kaminska B., Lifschitz C. (2015). Small bowel bacterial overgrowth associated with persistence of abdominal symptoms in children treated with a proton pump inhibitor. *Journal of Pediatrics*.

[22] Marchisio P., Claut L., Rognoni A. (2003). Differences in nasopharyngeal bacterial flora in children with nonsevere recurrent acute otitis media and chronic otitis media with effusion: implications for management. *The Pediatric Infectious Disease Journal*.

[23] Lima J. J., Lang J. E., Mougey E. B. (2013). Association of CYP2C19 polymorphisms and lansoprazole-associated respiratory adverse effects in children. *Journal of Pediatrics*.

[24] Ward R. M., Kearns G. L. (2013). Proton pump inhibitors in pediatrics. *Pediatric Drugs*.

[25] de la Coba Ortiz C., Argüelles Arias F., Martín de Argila de Prados C. (2016). Proton-pump inhibitors adverse effects: a review of the evidence and position statement by the Sociedad Española de Patología Digestiva. *Revista Española de Enfermedades Digestivas*.

[26] Pearson F., Mann K. D., Rees A., Davis A., Pearce M. S. (2015). The effect of childhood infection on hearing function at age 61 to 63 years in the newcastle thousand families study. *Ear and Hearing*.

[27] Aarhus L., Tambs K., Kvestad E., Engdahl B. (2015). Childhood otitis media. *Ear and Hearing*.

[28] Tian C., Harris B. S., Johnson K. R., Heymann D. (2016). Ectopic mineralization and conductive hearing loss in enpp1asj mutant mice, a new model for otitis media and tympanosclerosis. *PLoS ONE*.

[29] Ghebremariam Y. T., Lependu P., Lee J. C. (2014). Proton pump inhibitors and cardiovascular risk. *Circulation*.

[30] Ghebremariam Y. T., LePendu P., Lee J. C. (2014). Response to Letters Regarding Article, “unexpected effect of proton pump inhibitors: elevation of the cardiovascular risk factor asymmetric dimethylarginine”. *Circulation*.

[31] Sherwood M. W., Melloni C., Jones W. S., Washam J. B., Hasselblad V., Dolor R. J. (2015). Individual proton pump inhibitors and outcomes in patients with coronary artery disease on dual antiplatelet therapy: a systematic review. *Journal of the American Heart Association*.

[32] Shah N. H., Lependu P., Bauer-Mehren A. (2015). Proton pump inhibitor usage and the risk of myocardial infarction in the general population. *PLoS ONE*.

[33] Abraham N. S. (2012). Proton pump inhibitors. *Current Opinion in Gastroenterology*.

[34] Sukhovershin R. A., Cooke J. P. (2016). How may proton pump inhibitors impair cardiovascular health?. *American Journal of Cardiovascular Drugs*.

[35] Cooke J. P., Mont-Reynaud R., Tsao P. S., Maxwell A. J., Ignarro L. J. (2000). Nitric Oxide and Vascular Disease. *Nitric Oxide: Biology and Chemistry*.

[36] Sukhovershin R. A., Yepuri G., Ghebremariam Y. T. (2015). Endothelium-derived nitric oxide as an antiatherogenic mechanism: implications for therapy. *Methodist DeBakey Cardiovascular Journal*.

[37] Candipan R. C., Wang B., Buitrago R., Tsao P. S., Cooke J. P. (1996). Regression or Progression. *Arteriosclerosis, Thrombosis, and Vascular Biology*.

[38] MacAllister R. J., Parry H., Kimoto M. (1996). Regulation of nitric oxide synthesis by dimethylarginine dimethylaminohydrolase. *British Journal of Pharmacology*.

[39] Wang Z., Tang W. H. W., Cho L., Brennan D. M., Hazen S. L. (2009). Targeted metabolomic evaluation of arginine methylation and cardiovascular risks: potential mechanisms beyond nitric oxide synthase inhibition. *Arteriosclerosis, Thrombosis, and Vascular Biology*.

[40] Wilson A. M., Shin D. S., Weatherby C. (2010). Asymmetric dimethylarginine correlates with measures of disease severity, major adverse cardiovascular events and all-cause mortality in patients with peripheral arterial disease. *Vascular Medicine*.

[41] Lu T.-M., Chung M.-Y., Lin M.-W., Hsu C.-P., Lin S.-J. (2011). Plasma asymmetric dimethylarginine predicts death and major adverse cardiovascular events in individuals referred for coronary angiography. *International Journal of Cardiology*.

[42] Heinecke J. W., Kawamura M., Suzuki L., Chait A. (1993). Oxidation of low density lipoprotein by thiols: Superoxide-dependent and - independent mechanisms. *Journal of Lipid Research*.

[43] Cooke J. P., Ghebremariam Y. T. (2011). DDAH Says NO to ADMA. *Arteriosclerosis, Thrombosis, and Vascular Biology*.

[44] Ghebremariam Y. T., Lependu P., Lee J. C. (2013). Unexpected effect of proton pump inhibitors: elevation of the cardiovascular risk factor asymmetric dimethylarginine. *Circulation*.

[45] Cooke J. P., Tsao P. S. (1993). Cytoprotective effects of nitric oxide.. *Circulation*.

[46] Maggio M., Corsonello A., Lattanzio F., Cattabiani C., Lauretani F., Ceda G. (2012). Proton pump inhibitors and risk of 1-year mortality and rehospitalization in older patients discharged from acute care hospital. *European Geriatric Medicine*.

[47] Stühlinger M. C., Oka R. K., Graf E. E. (2003). Endothelial dysfunction induced by hyperhomocyst(e)inemia. *Circulation*.

[48] Lentz S. R. (2005). Mechanisms of homocysteine-induced atherothrombosis. *Journal of Thrombosis and Haemostasis*.

[49] Nygård O., Vollset S. E., Refsum H., Brattström L., Ueland P. M. (1999). Total homocysteine and cardiovascular disease. *Journal of Internal Medicine*.

[50] Welch G. N., Loscalzo J. (1998). Homocysteine and atherothrombosis. *The New England Journal of Medicine*.

[51] Nishinaga M., Ozawa T., Shimada K. (1993). Homocysteine, a thrombogenic agent, suppresses anticoagulant heparan sulfate expression in cultured porcine aortic endothelial cells. *The Journal of Clinical Investigation*.

[52] Rodgers G., Conn M. (1990). Homocysteine, an atherogenic stimulus, reduces protein C activation by arterial and venous endothelial cells. *Blood*.

[53] McColl K. E. (2009). Effect of Proton Pump Inhibitors on Vitamins and Iron. *American Journal of Gastroenterology*.

[54] Lundberg J. O., Weitzberg E., Lundberg J. M., Alving K. (1994). Intragastric nitric oxide production in humans: measurements in expelled air. *Gut*.

[55] Pinheiro L. C., Montenegro M. F., Amaral J. H., Ferreira G. C., Oliveira A. M., Tanus-Santos J. E. (2012). Increase in gastric pH reduces hypotensive effect of oral sodium nitrite in rats. *Free Radical Biology & Medicine*.

[56] Ho P. M. (2009). Adverse Outcomes Associated With Use of Proton Pump Inhibitors and Clopidogrel—Reply. *Journal of the American Medical Association*.

[57] Bhatt D. L., Cryer B. L., Contant C. F. (2010). Clopidogrel with or without omeprazole in coronary artery disease. *The New England Journal of Medicine*.

[58] Juurlink D. N., Gomes T., Ko D. T. (2009). A population-based study of the drug interaction between proton pump inhibitors and clopidogrel. *Canadian Medical Association Journal*.

[59] Charlot M. (2010). Proton-Pump Inhibitors Are Associated With Increased Cardiovascular Risk Independent of Clopidogrel Use. *Annals of Internal Medicine*.

[60] Schmidt M., Johansen M. B., Robertson D. J. (2012). Concomitant use of clopidogrel and proton pump inhibitors is not associated with major adverse cardiovascular events following coronary stent implantation. *Alimentary Pharmacology & Therapeutics*.

[61] Juel J., Pareek M., Jensen S. (2012). The Clopidogrel-PPI Interaction: An Updated Mini-Review. *Current Vascular Pharmacology*.

[62] D’Ugo E., Rossi S., De Caterina R. (2014). Proton pump inhibitors and clopidogrel: an association to avoid?. *Internal and Emergency Medicine*.

[63] Miller J. M., Ren T.-Y., Nuttall A. L. (1995). Studies of inner ear blood flow in animals and human beings. *Otolaryngology—Head and Neck Surgery*.

[64] Nakashima T., Naganawa S., Sone M. (2003). Disorders of cochlear blood flow. *Brain Research Reviews*.

[65] Erkan A., Beriat G., Ekici B., Doğan C., Kocatürk S., Töre H. (2015). Link between angiographic extent and severity of coronary artery disease and degree of sensorineural hearing lossBeziehung zwischen Ausmaß und Schweregrad der koronaren Herzkrankheit in der Angiographie und Grad der sensorineuralen Schwerhörig. *Herz*.

[66] Nomiya R., Nomiya S., Kariya S. (2008). Generalized Arteriosclerosis and Changes of the Cochlea in Young Adults. *Otology & Neurotology*.

[67] Mazurek B., Winter E., Fuchs J., Haupt H., Gross J. (2003). Susceptibility of the hair cells of the newborn rat cochlea to hypoxia and ischemia. *Hearing Research*.

[68] Pirodda A., Brandolini C., Borghi C. (2016). The influence of systemic circulation on hearing: The reliability of a different impact of microcirculatory defects and atherosclerosis. *Medical Hypotheses*.

[69] Paul W. F., Haughey B. H., Robbins K. T. (2014). *Cummings Otolaryngology - Head and Neck Surgery*.

[70] Badiola N., Alcalde V., Pujol A. (2013). The Proton-Pump Inhibitor Lansoprazole Enhances Amyloid Beta Production. *PLoS ONE*.

[71] Vaezi M. F., Yang Y., Howden C. W. (2017). Complications of Proton Pump Inhibitor Therapy. *Gastroenterology*.

[72] Stonnington C. M., Chen K., Lee W. (2014). Fibrillar amyloid correlates of preclinical cognitive decline. *Alzheimer's & Dementia*.

[73] Abbasi J. (2018). Plasma Biomarkers Predict Brain Amyloid-*β* Burden. *Journal of the American Medical Association*.

[74] Yang J., Dehom S., Sanders S., Murry T., Krishna P., Crawley B. K. (2018). Treating laryngopharyngeal reflux: Evaluation of an anti-reflux program with comparison to medications. *American Journal of Otolaryngology-Head and Neck Medicine and Surgery*.

[75] Lemajić-Komazec S., Abenavoli L. (2018). Iron deficiency anemia and hearing loss. *International Journal of Pediatric Otorhinolaryngology*.

[76] Chapman D. B., Rees C. J., Lippert D., Sataloff R. T., Wright S. C. (2011). Adverse Effects of Long-Term Proton Pump Inhibitor Use: A Review for the Otolaryngologist. *Journal of Voice*.

[77] Lecain E., Robert J., Thomas A., Tran Ba Huy P. (2000). Gastric proton pump is expressed in the inner ear and choroid plexus of the rat. *Hearing Research*.

[78] Shibata T., Hibino H., Doi K., Suzuki T., Hisa Y., Kurachi Y. (2006). Gastric type H + ,K + -ATPase in the cochlear lateral wall is critically involved in formation of the endocochlear potential. *American Journal of Physiology-Cell Physiology*.

[79] Mutschler E., Drozdzik M., Kocic I. (2018). *Mutschler farmakologia i toksykologia: Podrecznik*.

[80] Sone M., Kato T., Nakashima T. (2013). Current Concepts of Otitis Media in Adults as a Reflux-Related Disease. *Otology & Neurotology*.

[81] Schilder A. G., Chonmaitree T., Cripps A. W. (2016). Otitis media. *Nature Reviews Disease Primers*.

[82] McCoul E. D., Goldstein N. A., Koliskor B., Weedon J., Jackson A., Goldsmith A. J. (2011). A Prospective Study of the Effect of Gastroesophageal Reflux Disease Treatment on Children With Otitis Media. *Archives of Otolaryngology–Head & Neck Surgery*.

[83] Lin B. M., Curhan S. G., Wang M. (2017). Prospective Study of Gastroesophageal Reflux, Use of Proton Pump Inhibitors and H2-Receptor Antagonists, and Risk of Hearing Loss. *Ear and Hearing*.

